# Quantum control operations with fuzzy evolution trajectories based on polyharmonic magnetic fields

**DOI:** 10.1038/s41598-020-79309-8

**Published:** 2020-12-17

**Authors:** Jesús Fuentes

**Affiliations:** División de Ciencias e Ingenierías, Departamento de Física, Universidad de Guanajuato, Loma del Bosque 103, 37150 León, Guanajuato Mexico

**Keywords:** Physics, Quantum physics, Quantum mechanics, Single photons and quantum effects

## Abstract

We explore a class of quantum control operations based on a wide family of harmonic magnetic fields that vary softly in time. Depending on the magnetic field amplitudes taking part, these control operations can produce either squeezing or loop (orbit) effects, and even parametric resonances, on the canonical variables. For these purposes we focus our attention on the evolution of observables whose dynamical picture is ascribed to a quadratic Hamiltonian that depends explicitly on time. In the first part of this work we survey such operations in terms of biharmonic magnetic fields. The dynamical analysis is simplified using a stability diagram in the amplitude space, where the points of each region will characterise a specific control operation. We discuss how the evolution loop effects are formed by fuzzy (non-commutative) trajectories that can be closed or open, in the latter case, even hiding some features that can be used to manipulate the operational time. In the second part, we generalise the case of biharmonic fields and translate the discussion to the case of polyharmonic fields. Using elementary properties of the Toeplitz matrices, we can derive exact solutions of the problem in a symmetric evolution interval, leading to the temporal profile of those magnetic fields suitable to achieve specific control operations. Some of the resulting fuzzy orbits can be destroyed by the influence of external forces, while others simply remain stable.

## Introduction

A typical quantum control programme consists of a setup of oscillating external fields irradiating a micro-object (qubit) confined in a very small cavity either to induce a class of driven motion or to manoeuvre its degrees of freedom toward a special configuration. One shall be particularly cautious with some other details that cannot be disregarded, in that the settled presence of environmental noise, the radiative pollution or the emergence of electric shocks, amongst other issues, could result in an inoperative control protocol. Then, how to achieve the desired effects, at least averagely, in a simple but realisable way?

Customarily, some of these drawbacks can be successfully circumvented if a reasonable approximation is considered. There are control techniques based on magnetic nuclear resonance^1,2^, that accomplish acceptable results even if one ignores the intrinsic inhomogeneities as well as any electrical component conveyed by the sequence of variable magnetic fields, although beforehand the approximation relies on the quality of the generated magnetic pulses. Other schemes addressed to systems with discrete spectrum^3^ can suppress unwanted effects if the pulses of electrical forces depend only on the temporal domain. It is also the case for the effective description of an atom that moves across an optical device and interacts with a photon, which can be represented as an oscillating electric field^4,5^ as long as the dimensions of the cavity are small enough such that the approximation is not drastically far from the accurate description.

Nonetheless, whenever the particle is not strictly localised, another class of control techniques shall be considered. Think about the motion of an electron, in an unbounded state, that is controlled with a protocol of simple Rabi rotations. Hence, the implementation of ample cavities would be necessary due to the electron’s free propagation. These operations are usually implemented via ion traps^6–9^ or optical devices^10,11^, and receive special attention in a variety of applications where the unbounded motion shall be guided in a particular way, e.g. particle accelerators.

The interest in this kind of problems has motivated a number of works not limited to ion traps, but also in the realm of squeezed states^12^—useful to produce efficient non-demolition measurements^13,14^. Although the method is, once again, based on an approximation. It basically relies on the application of harmonic oscillator potentials with time-dependent frequencies, producing an effective oscillator field inside the walls of a hyperbolic structure, which is in turn connected to a pulsating electric potential^6,15^.

Our discussion below is addressed to these types of control problems, focusing our attention on time-dependent magnetic fields that are modulated in intensity by way of control currents. We shall design magnetic field pulses whose temporal profile is such that a charged particle under their influence evolves in time with either a stable (ion traps) or unstable (squeezing) motion, even neither of them, which yields a particular operation that produces parametric resonance.

Whenever the magnetic pulse is smooth enough, we will be allowed to look for exact solutions of the evolution problem. However, the application of soft operations will not be absent from very tiny discontinuities in the evolution process^16^ due to the concatenation of pulses. Indeed the presence of control currents produces a circular electric field enclosing the magnetic field, which could yield a sudden electrical jump. In our proposal, such discontinuities will be partially avoided by observing that the unitary matrices of evolution satisfy a simple Toeplitz algebra.

Even more, these unitary matrices of evolution truly reproduces the evolution loop phenomenon within the structure of fuzzy orbits^17,18^, which makes the particle to return to its initial values after a certain period of time (i.e. the interval of operation), albeit according to the type of motion taking part the fuzzy orbits can be open or broken. From our viewpoint, these orbits have a semiclassical character in that we are to consider only periodic, quadratic Hamiltonians, yet the loop phenomenon in itself also arises in problems with non-quadratic Hamiltonians^19,20^. This interesting aspect entices a variety of quantum control applications^16,21,22^ as well as some theoretical aspects in quantum tomography^23–25^.

In agreement with causality, the change in the field intensity due to time-varying currents will not be registered instantaneously in every part of the laboratory, instead the effects will be sensed depending on the dimensions of the laboratory and on the velocity of the signals. In that regard, an accurate treatment of the problem should include the retarded field effects, nevertheless, for simplicity we have restricted our analysis to the non-relativistic regime neglecting the amount of time in which the fields propagate. Our simplification deserves special attention for a practical implementation, and we shall survey the consequences as well.

For a self-contained discussion, let us start summarising the known facts^26^. Consider a spinless particle of charge *e* and fixed mass *m* moving in a uniform magnetic field ${\mathbf {B}}({\mathbf {x}}) \in {\mathbb {R}}^3$, generated by a class of vector potential ${\mathbf {A}}({\mathbf {x}})$ through the relation ${\mathbf {B}}({\mathbf {x}})=\nabla \times {\mathbf {A}}({\mathbf {x}})$. In particular, if the magnetic field is homogeneous a possible choice of ${\mathbf {A}}({\mathbf {x}})$ is

1 $$\begin{aligned} {\mathbf {A}}({\mathbf {x}})=\frac{1}{2}{\mathbf {B}}({\mathbf {x}})\times {\mathbf {x}}, \end{aligned}$$

where ${\mathbf {B}}({\mathbf {x}})$ will be aligned in the direction *Oz*, for the sake of convenience. As well, it is worthy of note how this gauge condition does not define uniquely ${\mathbf {A}}({\mathbf {x}})$ when ${\mathbf {B}}({\mathbf {x}})$ is given.

Let us start with a time-independent, quadratic Hamiltonian of the form:

2 $$\begin{aligned} H=\frac{m}{2}{\mathbf {V}}^2, \end{aligned}$$

where ${\mathbf {V}} = \frac{1}{m}\left[ {\mathbf {P}} - \frac{e}{c}{\mathbf {A}}({\mathbf {X}})\right]$ is the operator that accounts for the velocity of the particle and ${\mathbf {P}}$ is the operator of momentum. At this level ${\mathbf {A}}({\mathbf {X}})$ is also an operator and depends on the observable of position ${\mathbf {X}}$. Besides, we see that the observables ${\mathbf {X}}$ and ${\mathbf {P}}$ obey the usual commutation relations $[{\mathbf {X}}_j,{\mathbf {P}}_k]=i\hbar \delta _{jk}$, $[{\mathbf {X}}_j,{\mathbf {X}}_k]=0$ and $[{\mathbf {P}}_j,{\mathbf {P}}_k]=0$. Whereas the components of the operator ${\mathbf {V}}$ satisfy $[{\mathbf {V}}_j,{\mathbf {V}}_k]=\frac{ie\hbar }{m^2c}\epsilon _{jkl}{\mathbf {B}}_l({\mathbf {X}})$, where $\epsilon _{jkl}$ is 1 (-1) if *j*, *k*, *l* is and even (odd) permutation of 1,2,3, otherwise it is zero. Likewise, the commutation relations between the components of ${\mathbf {X}}$ and ${\mathbf {V}}$ are effortlessly obtained given that ${\mathbf {A}}({\mathbf {X}})$ commutes with ${\mathbf {X}}_j$, it follows that $[{\mathbf {X}}_j,{\mathbf {V}}_k]=\frac{i}{m}\hbar \delta _{jk}$.

There are special features that shall not escape attention. Unlike the classical problem where the trajectory described by the particle is fully localisable, in the quantum analogue a type of abstract space localisations will enter into the picture. The quantum particle will describe a trajectory whose centre $({\bar{X}},{\bar{Y}})$ is a fuzzy point, i.e. it is not fully localisable given that the operators associated with its position do not commute $[{\bar{X}},{\bar{Y}}]\ne 0$ —even either commuting or not there is nothing at that point, despite its geometric origin^27,28^. For instance, take the gauge (1), then the Hamiltonian will separate into two components $H=H_\perp + H_\parallel$, the transversal motion (the one of our interest) will occur in the *xOy* plane whereas the parallel motion will be aligned to *Oz*. The components of the operator ${\mathbf {V}}$ are:

3 $$\begin{aligned} \begin{aligned} V_x&=\frac{P_x}{m} + \frac{\omega _c}{2}Y\\ V_y&=\frac{P_y}{m} - \frac{\omega _c}{2}X\\ V_z&=\frac{P_z}{m}, \end{aligned} \end{aligned}$$

where $\omega _c=\frac{e}{mc}B$ is the cyclotron frequency and $B=|{\mathbf {B}}({\mathbf {X}})|$. The respective rotation centre results in a fuzzy point with coordinates:

4 $$\begin{aligned} {\bar{X}}=X+\frac{1}{\omega _c}V_y, \quad {\bar{Y}}=Y-\frac{1}{\omega _c}V_x, \end{aligned}$$

that satisfies the commutation relation $[{\bar{X}},{\bar{X}}]=-\frac{i\hbar }{m\omega _c}$. Note that if we define $\rho ^2=(X-{\bar{X}})^2+(Y-{\bar{Y}})^2$, then $\pi \rho ^2=\frac{2\pi }{m\omega _c^2}H$ can be interpreted as a fuzzy surface spanned by the transversal orbits. One can conclude that the surface $\pi \rho ^2$ is a constant of the motion, yet it cannot change continuously^29^ since it is quantised. This fact has a number of applications in the framework of loop quantum gravity^30,31^, for instance.

Indeed the fuzzy centres are not limited to appear in quantum Hall-effect models^32^ under the influence of a fixed magnetic field, but still occur in time-dependent problems. In our case, we will discuss how the time-varying magnetic operations become responsible for a number of noncommutative aspects supported on closed or open semiclassical trajectories.

In turn, we are to define the time-dependent version of the Hamiltonian (2), actually it can be written in a similar fashion but considering a magnitude-controlled current *I*(*t*) that modulates ${\mathbf {A}}({\mathbf {X}})$ and ${\mathbf {B}}({\mathbf {X}})$, in this way their corresponding time-dependent versions are written as ${\mathbf {A}}(t,{\mathbf {X}}) = I(t){\mathbf {A}}({\mathbf {X}})$ and ${\mathbf {B}}(t,{\mathbf {X}}) = I(t){\mathbf {B}}({\mathbf {X}})$. Such definitions imply that the time-dependent, quadratic Hamiltonian describing the motion of the non-relativistic, spinless, charged particle reads as^33^:

5 $$\begin{aligned} H(t)=\frac{m}{2}{\mathbf {V}}^2(t)=\frac{1}{2m}\left[ {\mathbf {P}}^2-\frac{e}{c}{\mathbf {A}}(t,{\mathbf {X}})\right] ^2 = \underbrace{\vphantom{\left[\left(\frac{eB(t)}{2c}\right)^2\right]}\frac{1}{2m}\left[ P_x^2 + P_y^2 + \left( \frac{eB(t)}{2c}\right) ^2(X^2+Y^2)\right] }_{H_\text {osc}(t)} - \underbrace{\frac{eB(t)}{2mc} L_z}_{H_\text {rot}(t)} + \underbrace{\vphantom{\left[\left(\frac{eB(t)}{2c}\right)^2\right]} \frac{1}{2m}P_z^2}_{H_\parallel }. \end{aligned}$$

Given that $H_\parallel$ leads to the well known free particle eigenfunction $\frac{1}{\sqrt{2\pi \hbar }}e^{ip_z z /\hbar }$, we are to separate this part from our analysis, and only pay attention to $H_\perp (t)=H_\text {osc}(t) + H_\text {rot}(t)$, where the oscillatory term $H_\text {osc}(t)$ is truly equivalent to a two dimensional harmonic oscillator, whereas the easily integrable term $H_\text {rot}(t)$ represents the rotations caused by $L_z$—the component of the angular momentum projected onto *Oz*—which is a conserved quantity. The former picture resembles, for example, a cylinder whose symmetry axis is parallel to *Oz* and carries a homogeneous current density on its surface.

To proceed it is convenient to express the perpendicular terms in (Eq. 5) in dimensionless variables, in order, we symbolise with $T=\frac{2\pi }{\omega }$ the time scale or period of operation. Our new variables become:

6 $$\begin{aligned} t\rightarrow \frac{t}{T},\qquad {\mathbf {P}}\rightarrow \sqrt{\frac{T}{\hbar m}}{\mathbf {P}},\qquad {\mathbf {X}}\rightarrow \sqrt{\frac{m}{\hbar T}}{\mathbf {X}}, \end{aligned}$$

in consequence the intensity of the oscillatory field is written as:

7 $$\begin{aligned} \beta (t)=\frac{eTB(t)}{2mc}. \end{aligned}$$

The function $\beta (t)$ is, in some extent, the cornerstone of our study: We shall implement a continuous, real function that solves the evolution problem. One is much in need to be specially careful with this task, for if $\beta (t)$ is bounded and piecewise continuous, the evolution of ${\mathbf {X}}$ and ${\mathbf {P}}$ will be continuous as well, although the same is not true for the components of the kinetic momentum provided there are sudden jumps that can be interpreted as electric shocks^34,35^ ${\mathbf {E}}=-\frac{e}{c} \frac{\partial }{\partial t}{\mathbf {A}}(t,{\mathbf {X}})$.

The substitution of Eqs. (6) and (7) into the perpendicular terms of the Hamiltonian (Eq. 5) yields the simplified, dimensionless expression:

8 $$\begin{aligned} H_\perp (t)=\underbrace{\frac{1}{2}\left( {\mathbf {P}}^2 + \beta ^2(t){\mathbf {X}}^2\right) }_{H_\text {osc}(t)} - \underbrace{ \vphantom{\frac{1}{2}} \beta (t)L_z}_{H_\text {rot}(t)}, \end{aligned}$$

in the special case that *B*(*t*) oscillates periodically (a regular Floquet problem) with frequency $\omega$, the substitution $H_\perp (t\omega )\rightarrow \frac{H_\perp (t)}{\hbar \omega }$ corresponds also to a dimensionless representation. One must be aware that in such case the stability regions of the motion described by the older Hamiltonian will no be valid for the dimensionless Hamiltonian any more.

Even though our Report is wholly concerned with magnetic operations, we would like to remark that despite the elementary and minimalistic structure of the Hamiltonian (Eq. 8), it can also describe the evolution of charged particles in hyperbolically shaped Paul’s traps^6^ of radius $r_0$. Under such circumstances, the time-dependent electric potentials of the type $\Phi (t,{\mathbf {X}})=\frac{e\phi (t)}{2r_0^2}\left( X^2+Y^2-2Z^2\right)$ or $\Phi (t,{\mathbf {X}})=\frac{e\phi (t)}{2r_0^2}\left( X^2-Y^2\right)$ define a modulated intensity $\beta (t)=\frac{eT^2\phi (t)}{mr_0^2}$. One immediately notes that the evolution problem can be handled in a similar fashion to the magnetic problem—in both cases yielding evolution matrices $u(t,t_0)$ identical for classical and quantum dynamics.

Below we are to solve the evolution problem described by Eq. (8). The already studied biharmonic amplitudes^9,34^ $\beta (t)$ occupy the first part of our survey, but we shall reformulate the stability map where the magnetic control operations are comprised. In the second part, we shall generalise the biharmonic approach in terms of polyharmonic amplitudes $\beta (t)$, that at the same time play the role of exact solutions. For this case we cannot generate a stability map such as the Strutt diagram that governs the harmonic and biharmonic cases. The systematic classification of fuzzy orbits in the polyharmonic case remains open.

## Results

We start with the operator equations satisfied by the unitary evolution operators $U(t,t_0)$:

9 $$\begin{aligned} \frac{d}{dt}U(t,t_0)=-iH(t)U(t,t_0), \quad \frac{d}{dt_0}U(t,t_0)=iU(t,t_0)H(t_0), \quad U(t_0,t_0)=\mathbbm{1}, \end{aligned}$$

nonetheless, for convenience and without loss of generality, we chose deliberately $t_0=0$ in order to introduce the abbreviations $u(t)=u(t,0)$ and $U(t)=U(t,0)$.

As well let us represent with the column-vector ${\mathbf {Q}}$ of dimension 2*N* the set of ordered pairs of observables ${\mathbf {X}}$ and ${\mathbf {P}}$ as ${\mathbf {Q}}=(Q_1,\ldots ,Q_{2N})^T=(({\mathbf {X}},{\mathbf {P}})_1,\ldots ,({\mathbf {X}},{\mathbf {P}})_{2N})^T$ (the notation $A^T$ indicates the transpose of the matrix *A*). In our particular case ${\mathbf {Q}}=(X,P_x,Y,P_y)^T$, recall that we are exclusively interested in the motion projected into the plane *xOy*, described by the Hamiltonian $H_\perp (t)$. Then a set of time-dependent observables ${\mathbf {X}}(t)$ and ${\mathbf {P}}(t)$ in the Heisenberg picture will evolve as a linear combination of their initial values ${\mathbf {X}}$ and ${\mathbf {P}}$ according to the rule:

10 $$\begin{aligned} {\mathbf {Q}}(t)=U^\dagger (t){\mathbf {Q}}U(t)=u(t){\mathbf {Q}}, \end{aligned}$$

where *u*(*t*) is a $2N\times 2N$ time evolution matrix that determines uniquely the evolution operator *U*(*t*), and vice versa, iff ${\mathbf {Q}}$ spans a complete set of observables in a Hilbert space ${\mathbbm {H}}$.

We can write down the *j*-th coordinate of the fuzzy centre (Eq. 4):

11 $$\begin{aligned} {\bar{X}}_j(t)={\bar{u}}_{j1}X_1+{\bar{u}}_{j2}P_1+\cdots +{\bar{u}}_{j,2N-1}X_{N}+{\bar{u}}_{j,2N}P_{N}, \quad (X_1,X_2,X_3)=(X,Y,Z), \quad {\bar{u}}_{jk}=\frac{1}{T}\int _0^Tdt\, u_{jk}(t). \end{aligned}$$

The direct application of the matrix *u*(*t*) to the initial vector ${\mathbf {Q}}$ will generate the whole trajectory of motion. Furthermore, since $H_\perp (t)$ is quadratic in its variables, the evolution operator *U*(*t*) will be the same in the classical and quantum regimes. Thus, the classical motion trajectories *U*(*t*) will be analogously interpreted in the quantum case, where, the mean position $\langle {\mathbf {X}}\rangle$ will evolve according to $i\hbar \frac{d}{dt}\langle {\mathbf {X}}\rangle =\langle [{\mathbf {X}},H_\perp (t)]\rangle$ (Ehrenfest’s theorem).

Note that if two unitary operators $U_1(t)$ and $U_2(t)$ lead to the transformation

$$\begin{aligned} U_1^\dagger (t){\mathbf {Q}}U_1(t)=U_2^\dagger (t){\mathbf {Q}}U_2(t) \quad \text {then} \quad U_2(t)U_1^\dagger (t){\mathbf {Q}}={\mathbf {Q}}U_2(t)U_1^\dagger (t) \end{aligned}$$

and $U_2(t)U_1^\dagger (t)$ commutes simultaneously with any function depending on ${\mathbf {X}}$ and ${\mathbf {P}}$. Moreover, the observables ${\mathbf {X}}$ and ${\mathbf {P}}$ generate an irreducible algebra in $L^2({\mathbbm {R}})$, therefore $U_1(t)U_2^\dagger (t)$ must be a phase factor provided these are unitary operators. As a result we have $U_1(t)U_2^\dagger (t)=e^{i\varphi }$, or equivalently $U_1(t)=e^{i\varphi }U_2(t)$ where $\varphi$ is a real number. Such detail becomes relevant in quantum control problems, for if any two unitary operators differ by a phase factor, they generate the same transformation of quantum states, so both are equivalent in this sense.

The condition of evolution loop will be satisfied inasmuch as the whole set of observables ${\mathbf {Q}}$ return to their initial conditions after a time interval *T*. In consequence the analysis is considerably simplified if we assume that Eq. (8) possesses a periodic temporal dependence, such that $H_\perp (T+t)=H_\perp (t)$, that is a Floquet Hamiltonian. As well, the periodicity of Eq. (8) implies that $U(t)=U(T+t)$, concluding that any observable ${\mathbf {Q}}$ in the Heisenberg picture will evolve periodically even if it does not depend explicitly on time. It follows from Eq. (10) that ${\mathbf {Q}}(T+t)=U^\dagger (T+t){\mathbf {Q}}U(T+t)={\mathbf {Q}}(t)$, or for a fixed period of time evolution ${\mathbf {Q}}(T)=U^\dagger (T){\mathbf {Q}}U(T)={\mathbf {Q}}$, which means that the loop condition indicates $U(T)=e^{i\varphi }\mathbbm{1}$.

### Biharmonic fields

We shall now analyse the evolution loop trajectories (flowing inside of a solenoid with a time-varying, homogeneous current density on its surface) as well as the squeezing effects achieved by biharmonic oscillations of the type^34^:

12 $$\begin{aligned} \beta (t)=\beta _0+\beta _1\sin (\omega _1 t) + \beta _2 \sin (\omega _2 t), \quad \omega _1,\omega _2\in {\mathbb {R}}, \end{aligned}$$

where the selection of either $\beta _2=0$ or $\omega _2=0$ enables the harmonic case.

The temporal profile of $\beta (t)$ is not restricted to a particular form, in fact it can be quite arbitrary. Nevertheless any reasonable physical implementation of $\beta (t)$ shall preferably avoid any sudden jump such as the ones furnished by kicked protocols^16,21,34,35^, since the resulting electric delta shocks could compromise an even evolution. Accordingly, an alternative is to choose a sufficiently smooth pulse in terms of harmonic functions.

An interesting consequence of our cylindrical model is that the commutation relation $[H_\text {osc}(t),H_\text {rot}(t')]=0$ is invariably satisfied, thus the evolution operator *U*(*t*) can be split into two distinct components, $U(t)=U_\text {rot}(t)U_\text {osc}(t)$, describing the evolution around *Oz* and the evolution of the magnetic oscillator $U_\text {osc}(t)$, respectively, each one satisfying Eq. (9). The resulting evolution matrix *u*(*t*), therefore, will consist of two steps of evolution starting from the initial condition ${\mathbf {Q}}$ to ${\mathbf {Q}}_\text {osc}(t)$ to ${\mathbf {Q}}(t)$ unfolded by the consecutive application of the oscillatory and rotational evolutions, in that order.

The matrix of rotations $u_\text {rot}(t)$ will be generated by the operator $U_\text {rot}(t)$, which is straightforwardly integrated as

13 $$\begin{aligned} U_\text {rot}(t)=e^{-iL_z\int _0^tdt'\beta (t')}, \end{aligned}$$

whereas the matrix $u_\text {osc}(t)$ of dimension $4\times 4$ will be constructed from $U_\text {osc}(t)$. Fortunately, the integration of the oscillatory part can be further simplified due to $u_\text {osc}(t)$ can be reduced to a single matrix *h*(*t*) that will make each pair of observables $Q_x=(X,P_x)^T$ and $Q_y=(Y,P_y)^T$ to evolve simultaneously, i.e.

14 $$\begin{aligned} u_\text {osc}(t)=\begin{pmatrix} h(t)&{} \\ &{} h(t)\end{pmatrix} \end{aligned}$$

will act at once in each of the subspaces spanned by $Q_x$ and $Q_y$. In that regard, for $Q_j$ with $j=x,y$, we have:

15 $$\begin{aligned} \frac{dh(t)}{dt}Q_j = iU_\text {osc}^\dagger (t)[H_\text {osc}(t), Q_j]U_\text {osc}(t)=\Lambda (t)U_\text {osc}^\dagger (t)Q_jU_\text {osc}(t)=\Lambda (t)h(t)Q_j, \end{aligned}$$

where

16 $$\begin{aligned} \Lambda (t)=\begin{pmatrix}0&1\\ \beta^2(t)&{}0\end{pmatrix}, \end{aligned}$$

concluding that *h*(*t*) simply obeys the differential equation:

17 $$\begin{aligned} \frac{dh(t)}{dt}=\Lambda (t)h(t), \quad h(0)=\mathbbm{1}_{2\times 2}, \end{aligned}$$

while the integration of this equation becomes standard for stationary fields $\beta (t)=\beta _0$, yielding solutions of the form $e^{\Lambda t}$, in general we want a computer to do the job.

Observe that the determinant of the symplectic matrix *h*(*t*) is an integral of the evolution in that $\text {det}(h(t))=1$. This attribute turns essential to classify the particle’s motion as well as to find exact solutions, as we shall show later.

The matrix *h*(*t*) is at the core of any evolution process generated by the class of periodic fields $\beta (T+t)=\beta (t)$, and we must discuss the ascribed dynamical features to such matrix. First, *h*(*t*) is symplectic, it has two eigenvalues $\lambda ^+,\lambda ^-$ such that $\lambda ^+=\frac{1}{\lambda ^-}$, hence the algebraic structure of *h*(*t*) is wholly determined by the scalar $\Sigma =\text {tr}(h(t))$, as suggested by the characteristic polynomial $D(\lambda )=\lambda ^2-\Sigma \lambda +1$ and its roots $\lambda ^\pm =\frac{1}{2}(\Sigma \pm \sqrt{\Delta })$, where $\Delta =\Sigma ^2-4$. We are granted, therefore, to classify the outperformed motion through $|\Sigma |$ into three categories^34^:

$$|\Sigma |<2$$.
The matrix *h*(*t*) has the eigenvalues $\lambda ^+=e^{+i\sigma }$ and $\lambda ^-=e^{-i\sigma }$, $\sigma \in [0,\frac{\pi }{2}]$. The motion is completely stable and restricted to an oscillatory evolution. The annihilation and creation operators, $a^-$ and $a^+$, can be obtained from the eigenvectors of *h*(*t*). Control operations of this type are useful in ion traps.
$$|\Sigma |=2$$.
The eigenvalues of *h*(*t*) are merely $\pm 1$, defining a separatrix between the stable and unstable regions. The motion described by field amplitudes in the threshold region might originate an effective parametric resonance, which supports a variety of interesting applications, e.g. a charged particle could be attracted by repulsive forces.
$$|\Sigma |>2$$.
The matrix *h*(*t*) has a pair of real eigenvalues, $\lambda ^+=e^{+\sigma }$ and $\lambda ^-=e^{-\sigma }$, $\sigma \in [0,\frac{\pi }{2}]$. This class of motion is unstable, producing a squeezing effect over the annihilation and creation operators $a^-$ and $a^+$, i.e. expanding $a^-$ at the cost of $a^+$, or conversely as well.

Despite the classification given above is entirely based on the Heisenberg’s evolution of canonical observables, regarding material particles, the parametric resonance region, $|\Sigma |=2$, can be understood as an analogue of the parametric amplification studied by Mollow and Glauber^36^ in the context of coherent photon states. Even though the trajectory picture commonly attracts the concern of many researches as for the design of ion traps.

**Figure 1:**
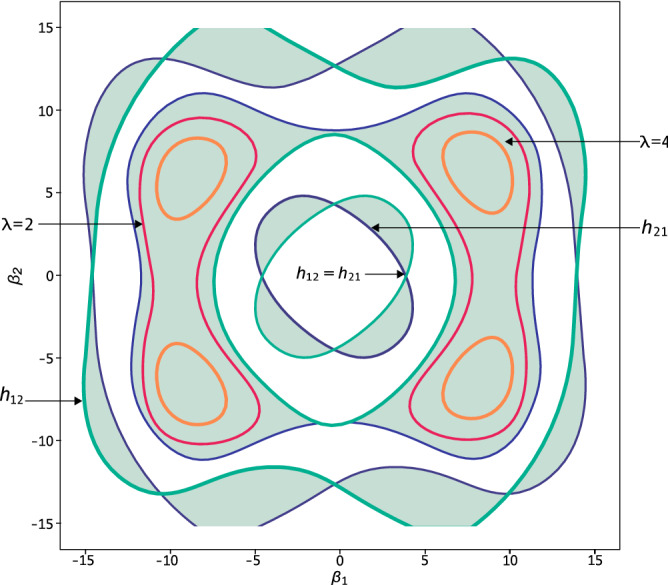
Strutt diagram of a biharmonic field (Eq. 12). The stability regions, $|\Sigma |<2$, correspond to the clear areas, whereas the instability regions, $|\Sigma |>2$, appear in colour. The threshold belts, $|\Sigma |=2$, can be tracked with the aid of the matrix entries $h_{11}$ or $h_{21}$. If both entries are simultaneously equal to zero we have a pure squeezing effect, otherwise, the squeezing operations can be generated by taking the points inside the coloured areas. That is the case of the tracked subregions of amplitudes that would achieve the effects of $h_{11}=\lambda =2$ or $h_{11}=\lambda =4$. A point $(\beta _1,\beta _2)$ lying in the clear areas could be used to produce a class of stable motion, showing a loop effect, such as the required for ion traps.

As an example, a well known ion trap is the radio-frequency, quadrupole device engineered by Paul^6^ in which the oscillating elastic forces $\beta (t)$ are written in terms of Mathieu functions. Indeed, the stability regions for such trap are simply identified from a Strutt map, useful in a variety of time-dependent problems with elastic potentials, e.g. tomography^23^.

We shall now illustrate how this diagram works, not only for ion traps, but for another quantum control operations. To this aim, we have devised a computer routine to integrate numerically Eq. (17) in terms of a biharmonic field (Eq. 12) with fixed $\beta _0=0$. A scanning process shall be meticulously undertaken, to track the different values of $|\Sigma |$ in the amplitude domain. We have chosen $\beta _1,\beta _2\in [-15,15]$ with angular frequencies $\omega _1=2\pi$, $\omega _2=4\pi$ and $t\in [0,1]$. As a result, we have drawn the biharmonic map in Fig. 1, where the three regions according to $|\Sigma |$, can be located in the amplitude space spanned by $\{\beta _1,\beta _2\}$, in other words, depending on the values of such amplitudes, a type of quantum control operation will be furnished.

**Figure 2:**
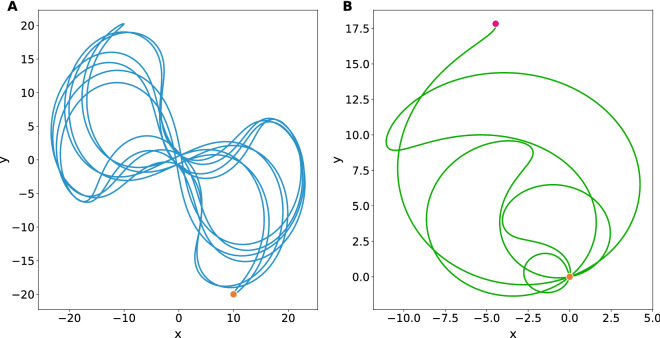
**(A)** Semiclassical closed trajectory in the plane *xOy* generated from the initial conditions $(x,y)=(10,-20)$ and $(p_x,p_y)=(-5,20)$. The corresponding dimensionless amplitudes of the elastic field are $\beta _1=\frac{\pi }{4}$ and $\beta _2=-10$ which lie in the region of stability as for Fig. 1. The loop closes after a period $T=6$. The orange dot represents its initial and final positions. **(B)** Broken loop of inverted free evolution. It takes the initial conditions $(x,y)=(0,0)$ and $(p_x,p_y)=(-5,20)$ with the amplitudes $\beta _1=-11.86$ and $\beta _2=-0.4$ which lie exactly in the separatrix between the stability and instability regions. For any operation that is realised with amplitudes of this kind, it will take a period of $T=2$ to complete the control operation. The orange (red) dot represents its initial (final) position.

First we are to survey the stable motion assured by the set of amplitudes $\{\beta _1,\beta _2\}$ lying in the clear areas of the map in Fig. 1. To explain how the evolution loops are originated, we have integrated Eq. (17) up to $T=6$ field periods, finding the closed trajectory shown in Fig. 2-A, whose corresponding dimensionless amplitudes are $\beta _1=\frac{\pi }{4}$ and $\beta _2=-10$. In this example, the particle starts its evolution with velocities $(p_x,p_y)=(-5,20)$ at the point $(x,y)=(10,-20)$ and finishes at the same point with velocities $(p_x,p_y)=(6.25,-2.41)$. Any evolution loop with symmetry under parity reflection, such as this one, will have a vanishing fuzzy point $({\bar{X}},{\bar{Y}})$ indicating immunity to the effect of external driven forces. In fact, whenever the elastic field $\beta (t)$ is biharmonic, the potential ${\mathbf {A}}(t,{\mathbf {X}})$ will evanesce at the beginning $t=0$ and at the end $t=T$ of the process, then the canonical momenta and the kinetic momenta coincide, simplifying the interpretation of the wave packet at these points.

The oscillatory motion is not restricted to circuits of evolution, it truly can be broken into open trajectories under some circumstances. These cases are particularly interesting since they involve a kind of free evolution embedded in the particle’s history of motion. We recall that in the Schrödinger picture the free evolution along a time interval $\tau$ corresponds to the operator $e^{-\frac{i\tau }{\hbar }\frac{{\mathbf {P}}^2}{2}}$, which transforms the canonical observables into $X_j\rightarrow X_j+\tau P_j$ and $P_j\rightarrow P_j$ via a matrix of evolution of the form (Eq. 14), although in this case $h(t) \rightarrow h(\tau )$ has the simple structure

18 $$\begin{aligned} h(\tau )=\begin{pmatrix}1&{}\tau \\ 0&{}1\end{pmatrix}, \end{aligned}$$

therefore, should $h(\tau )$ represents a segment of evolution loop the rest of the trajectory merely corresponds to the inverse operation $e^{+\frac{i\tau }{\hbar }\frac{{\mathbf {P}}^2}{2}}$, that is $h(-\tau )$, such that $h(\tau )h(-\tau )=\mathbbm{1}$. In other words, the action of $h(-\tau )$ reverts the effects produced by $h(\tau )$ over the set of observables, concentrating the wave packet instead of contributing to its spread across the interval of operation.

Such effects are produced by allowing the field $\beta (t)$ to take amplitudes $\{\beta _1,\beta _2\}$ living in the threshold region, $|\Sigma |=2$, allowing three classes of temporal manipulations in accordance with the effective time of operation $\tau$: (1) Accelerated free evolution, $\tau >T$, the system will get older faster than the actual time of operation; (2) Retarded free evolution, $0<\tau <T$, the system will experience a retarded progress in its temporal motion; (3) Inverted free evolution, $\tau <0$, the effect is generated by the successive application of two biharmonic periods $T=2$ as long as $\Sigma =-2$ (i.e. $h_{11}=h_{22}=-1$) hence $\tau =-2h_{12}$ with $h_{12}>0$.

As an example, let us consider the separatrix point $(\beta _1,\beta _2)=(-11.86,-0.4)$ on the map of Fig. 1. As for initial conditions we set $(x,y)=(0,0)$ and $(p_x,p_y)=(-5,20)$, thereon after two periods the particle will arrive at the point $(x,y)=(-4.47,17.85)$ with the same velocities, i.e. it will end at a distance $2\tau |v|$ behind its original location, see Fig. 2B.

Finally, we are to review the squeezing effects granted by those points pertaining to the coloured areas of the Strutt map, Fig. 1. Our comments ahead, however, are not addressed to the squeezed-states philosophy, rather we focus our attention on the source of the squeezing operations regarding the periodic-evolution model (Eq. 8).

Once again the symplectic structure of the matrix *h*(*T*), permits to simplify the analysis, and formulate the squeezing condition in terms of its eigenvalues as:

19 $$\begin{aligned} \lambda ^+\lambda ^-=1, \end{aligned}$$

fulfilled inside the region $|\Sigma |>2$. However, the time evolution outlined by Eq. (17) has to be calculated within a time interval where $\beta ^2(t)$ is antisymmetric around the interval centre, or the squeezing effects would not occur^37^. Moreover, depending on the actual value of $\Sigma$, there will be two possible squeezing transformations: the purely positive transformation for $\Sigma >2$ and the parity transformation for $\Sigma <2$. One can freely choose between these two options via the points $(\beta _1,\beta _2)$, based on the control purposes.

In the light of the condition (Eq. 19), it follows that the matrix elements $h_{12}$ and $h_{21}$ are equal to zero, and therefore any canonical observable *Q* will be transformed as:

20 $$\begin{aligned} Q'\rightarrow \lambda Q', \quad Q''\rightarrow \frac{1}{\lambda }Q'', \end{aligned}$$

which read as the amplification of $Q'$ at the cost of compressing $Q''$, or vice versa. Such transformations are, in fact, embedded in the eigenvectors of *h*(*T*).

It might also be noted that if a fuzzy point (Eq. 4) does not vanish, the squeezing operations will sense the influence of any external force but in an orthogonal direction to its exertion, otherwise the control operations become resistant to external perturbations, such as radiation pollution^38^.

Nonetheless, in general the kind of transformations (Eq. 20) regarding eigenvalues $x_j\rightarrow \lambda x_j$, $p_j\rightarrow \frac{1}{\lambda } p_j$ in intervals $[nT,(n+1)T]$, can only be produced at those separatrix points where $h_{12}=h_{21}=0$ is satisfied, namely at the intersection points of the matrix trajectories in the separatrix belt. The squeezing transformations (Eq. 20) are realisable if the amplitudes $\beta _1,\beta _2$ take values inside the unstable regions specified by the Strutt map. We have traced some of these points at which an intense squeezing $\lambda =h_{11}$ is achieved, particularly for $\lambda =2$ and $\lambda =4$. For instance, a biharmonic field with the amplitudes $(\beta _1,\beta _2)=(-10.3,-6.9)$ would attain a squeezing/amplification factor $\lambda =4$.

### Polyharmonic fields: exact operations

There exists a miscellany of methods to generate quantum control operations of the form (Eq. 20), amongst them the programmes of kicked (or discrete) pulses that disrupt a continuous evolution process^16,21,34^. Unfortunately, as we have already mentioned, this kind of operations result technically impractical leading to the necessity of a more method based on soft evolution operations. If the discrete pulses are replaced by smooth pulses, e.g., biharmonic fields $\beta (t)$, the imperfections are partially circumvented. Indeed, the design of such operations could be devised considering two different smooth pulses^35^, both in the stable region of the Strutt diagram, but demanding that the product between them belongs to the instability region, yet the successive application of both pulses makes the particle to absorb an amount of energy^39^, that shall be less than the difference between the neighbouring energy levels of the particle. How can we really avoid any sudden jump in the composition of quantum control operations? We shall not answer that question with utter certainty, rather we would like to sketch an attempt in the following discussion.

From our viewpoint, the composition approach consists of taking portions of the time evolution course described by the Hamiltonian (Eq. 8), with the exception that this time the elastic fields (Eq. 7) shall be transformed as $\beta ^2(t)\rightarrow \beta (t)$ to facilitate the quest of exact solutions. Hence, in the subspace of oscillations, the matrix *h*(*t*) becomes:

21 $$\begin{aligned} h(t)=\begin{pmatrix}\cos (\omega t) &{} \frac{1}{\omega }\sin (\omega t) \\ -\omega \sin (\omega t) &{} \cos (\omega t)\end{pmatrix}, \end{aligned}$$

noting that it adopts the form of a symplectic matrix of rotations.

In particular, for any $\omega t=\frac{n\pi }{2}$ (n is an integer) we obtain the squeezing transformations:

22 $$\begin{aligned} h\left( \frac{n\pi }{2\omega }\right) =\begin{pmatrix}0&{}\pm \frac{1}{\omega }\\ \mp \omega &{}0\end{pmatrix}, \end{aligned}$$

now, the successive application of two matrices of this type leads to:

23 $$\begin{aligned} h_\text {squeezing} = \begin{pmatrix}0&{}\pm \frac{1}{\omega _1}\\ \mp \omega _1&{}0\end{pmatrix}\begin{pmatrix}0&{}\pm \frac{1}{\omega _2}\\ \mp \omega _2&{}0\end{pmatrix}=\begin{pmatrix}\lambda &{}0\\ 0&{}\frac{1}{\lambda }\end{pmatrix}, \quad \lambda = -\frac{\omega _2}{\omega _1}, \quad \omega _1\ne \omega _2, \end{aligned}$$

leading again to the set of operations in Eq. (20) with the requirement of two distinct frequencies $\omega _1$ and $\omega _2$ at different times, say $t_1$ and $t_2$, such that $\omega _1t_1=\omega _2t_2=\frac{\pi }{2}$. The sudden jumps have not been removed already, for example, should the protocol be applied in the void background there would be at least three jumps involved: $0\rightarrow \omega _1\rightarrow \omega _2\rightarrow 0$. What if we translate this procedure to the continuum?

We have shown that the set of operations (Eq. 20) are actually generated by symplectic matrices *h*(*t*) with the property $h_{11}=h_{22}=\frac{1}{2}\Sigma$, i.e. *h*(*t*) has the form of a Toeplitz matrix. This agreeable property grants that if $\eta$ and $\xi$ are Toeplitz matrices, their anti-commutator algebra $\eta \xi + \xi \eta$, as well as their symmetric products $\eta \xi \eta$ and $\xi \eta \xi$, furnish matrices of the same class. It follows that the squeezing transformations (Eq. 20) can be built from the contribution of a big number of symmetric products between symplectic matrices $\eta (t)$ of the form (Eq. 21), each one at different time subintervals $t_j$ with a definite elastic field amplitude $\beta (t_j)$, where $j=0,1,2,\ldots$, namely:

24 $$\begin{aligned} h(t)= \eta (t_k) \cdots \eta (t_1) \eta (t_0) \eta (t_1) \cdots \eta (t_k), \end{aligned}$$

once again, conveying the property $h_{11}=h_{22}=\frac{1}{2}\Sigma$.

To obtain the continuous analogue we shall assume that the infinitesimal jumps *dh*(*t*) are assembled by the infinitesimal contributions $d\eta (t)=\Lambda (t)dt$, from the right to the left sides as in Eq. (24), each one depending on a symmetric field $\beta (t)=\beta (-t)$ around $t=0$, arriving at the matrix differential equation in the expanded interval $[-t,t]$:

25 $$\begin{aligned} \frac{dh(t)}{dt} = \Lambda (t)h(t)+h(t)\Lambda (t), \quad h(0)=\mathbbm{1}, \quad \Lambda (t)=\begin{pmatrix}0&{}1\\ -\beta(t)&{}0\end{pmatrix}, \end{aligned}$$

which is explicitly written as

26 $$\begin{aligned} \frac{dh(t)}{dt} = \begin{pmatrix}h_{21}-h_{12}\beta (t) &{} \Sigma \\ -\Sigma \beta (t) &{}h_{21}-h_{12}\beta (t)\end{pmatrix}=(h_{21}-h_{12}\beta (t))\mathbbm{1}+\Sigma \begin{pmatrix}0&{}1\\ -\beta(t) &{} 0\end{pmatrix}, \end{aligned}$$

nonetheless, the actual trajectory determined by the whole evolution process, requires the integration of Eq. (17) along an asymmetric interval, since the character of Eq. (25) is rather auxiliary as will be clear below. On top of that the anti-commutative structure of Eq. (25) defines a $\beta (t)$ in terms of a smooth enough, real function $\theta (t)$. What we are to discuss now is precisely how to obtain an exact solution of the inverse evolution problem.

To this aim, first note that the diagonal elements of *h*(*t*) satisfy $\frac{dh_{11}}{dt}=\frac{dh_{22}}{dt}=h_{21}-h_{12}\beta (t)$, but according to the initial condition $h_{11}=h_{22}=1$ at $t=0$, allowing to write

27 $$\begin{aligned} h_{11}=h_{22}=\frac{1}{2}\frac{d\theta (t)}{dt}, \end{aligned}$$

consistently, it follows that $\text {det}(h(t))=(\frac{1}{2}\frac{d\theta (t)}{dt})^2-h_{21}\theta (t)=1$, therefore:

28 $$\begin{aligned} h_{21}=\frac{\left[ \frac{1}{2}\frac{d\theta (t)}{dt}\right] ^2-1}{\theta (t)}, \end{aligned}$$

substituting into (27) and rearranging terms, yields $h_{12}\beta (t)=h_{21}-\frac{dh_{11}}{dt}$. Even more, since $\theta (t)=h_{12}$ it directly means $\frac{dh_{11}}{dt}=\frac{1}{2}\frac{d^2\theta (t)}{dt^2}$, in this fashion we get:

29 $$\begin{aligned} \beta (t)=-\frac{\frac{d^2\theta (t)}{dt^2}}{2\theta (t)}+\frac{\left[ \frac{1}{2}\frac{d\theta (t)}{dt}\right] ^2-1}{\theta ^2(t)}, \end{aligned}$$

one can immediately notice that the special case $\theta (t)=\frac{1}{\omega }\sin (2\omega t)$ conducts to the basic harmonic oscillator $\beta (t)=\omega ^2=\text {const.}$

Even without any specialised consideration on adiabatic invariants^40,41^, $\beta (t)$ as expressed in Eq. (29) is an exact solution of the inverse evolution problem for *h*(*t*) given a function $\theta (t)$, which is, in principle, arbitrary though constricted to satisfy non-trivial conditions at singular points. Nevertheless, for any asymmetric interval $[t_0,t]$ the dependence of *h*(*t*) on $\beta (t)$ must be determined by the integration of Eq. (17), as we have already shown for the case of biharmonic fields.

Some simple relations between $\beta (t)$ and $\theta (t)$ deserve observation. Any field amplitude given by Eq. (29) in a symmetric interval $[-t,t]$, is accomplished by a sufficiently smooth function $\theta (t)$ to assure continuity and differentiability. Specifically, at any point *t* where $\theta (t)=0$, there must be $\frac{d\theta (t)}{dt}=\pm 2$. As well, if $\theta (t)\ne 0$ but $\frac{d\theta (t)}{dt}=0$ then Eq. (25) becomes a matrix of squeezing transformations such as the one in Eq. (23). Moreover, if $\frac{d^3\theta (t)}{dt^3}=0$ it follows necessarily that $\frac{d\beta (t)}{dt}=0$.

**Figure 3:**
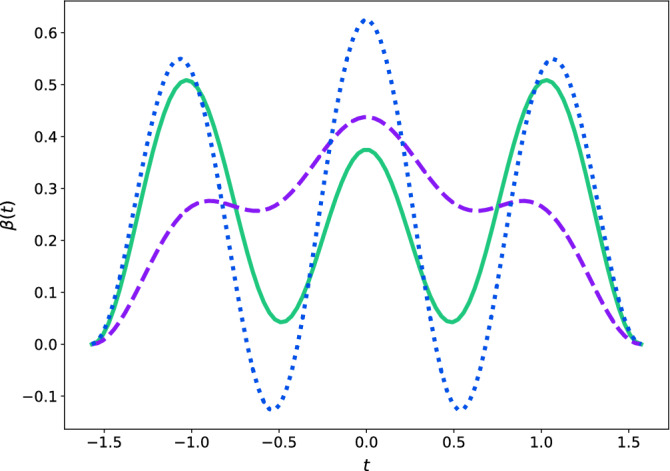
Temporal profile of three polyharmonic pulses $\beta (t)$ in the symmetric interval $\left[ -\frac{\pi }{2},\frac{\pi }{2}\right]$, with $b=2,c=-3$ (solid line), $b=\frac{9}{5}, c=-\frac{7}{2}$ (dashed), and $b=2,c=-5$ (dotted). The three pulses vanish at the endpoints of the interval, even though only the amplitudes in solid and dashed curves can be utilised to achieve squeezing transformations of the form (Eq. 20), since $\beta (t)>0$ in both cases, whereas the field amplitude depicted with a dotted curve could generate an evolution loop effect over the canonical variables—useful for ion traps, for instance—in that the matrix *h*(*t*) will fulfil the condition $|\Sigma |<2$ for full stability.

The only missing piece is the construction of $\theta (t)$. As we said, this function is truly arbitrary, although from an empirical viewpoint a natural choice would be a $\theta (t)$ in terms of harmonic functions to induce soft control operations and prevent the evolution process from any leap. Amongst the most elementary cases, let us consider the polyharmonic function

30 $$\begin{aligned} \theta (t) = a_1 \sin (\omega _1t) + a_3 \sin (\omega _3t) + a_5\sin (\omega _5t) + a_7 \sin (\omega _7t), \end{aligned}$$

since this function is antisymmetric around $t=0$ the corresponding $\beta (t)$ as for Eq. (29) will be symmetric around that point. Particularly, if we fix the frequencies as $\omega _1=1, \omega _3=3,\omega _5=5$ and $\omega _7=7$, at the endpoints of the symmetric interval $\left[ -\frac{\pi }{2},\frac{\pi }{2}\right]$ a matrix of squeezing (Eq. 23) will emerge with $h_{12}=\pm \omega =b$. Accordingly, the following initial conditions must be fulfilled:

31 $$\begin{aligned} \begin{aligned} \theta \left( \frac{\pi }{2}\right) = a_1 - a_3 + a_5 - a_7 = b,&\quad \theta '(0) = a_1 +3 a_3 + 5a_5 +7a_7 = 2,\\ \theta ''\left( \frac{\pi }{2}\right) = -a_1 +9 a_3 - 25 a_5 = -\frac{2}{b},&\quad \theta '''(0) = -a_1 -27 a_3 -125a_5 -343a_7 = c, \end{aligned} \end{aligned}$$

where $c\ne 0$ is a real parameter that we shall carefully fix to achieve the desired control operation. This set of rules gives additional information regarding the nontrivial relation between $\beta (t)$ and $\theta (t)$. The condition $\theta \left( \frac{\pi }{2}\right) =b$ determines the magnitude of the squeezing transformation depending on the whole trajectory, whereas $\theta '(0)=2$ endows $\beta (t)$ with non-singularity at $t=0$. In turn, the coefficients of $\theta (t)$ are:

32 $$\begin{aligned} \begin{aligned} a_1 = \frac{(105 b+c+58)b-10}{128 b},&\quad a_3 = -\frac{(35 b-c-74)b+2}{128 b}, \\ a_5 = \frac{18-(21 b+c-22)b}{384 b},&\quad a_7 = \frac{(15 b-c-26)b-6}{384 b}, \end{aligned} \end{aligned}$$

see the numerical examples of the generated pulses $\beta (t)$ in Fig. 3, in which we have selected $\beta \left( -\frac{\pi }{2}\right) =\beta \left( \frac{\pi }{2}\right) =0$.

Unlike the method based on biharmonic fields, where we could generate a map such as the one presented in Fig. 1, in the current case we are unable to pursue an analogue survey to scan the type of control operations produced by a set of amplitudes $\{\beta _1,\beta _2\}$. Even though, in the light of polyharmonic pulses the functions (Eq. 29) and (Eq. 30) truly offer additional information regarding the effects achieved at the end of the symmetric interval of operation: If $\beta (t)>0$ the magnetic field would induce a squeezing effect over the canonical observables in the concatenated intervals $\left[ -\frac{\pi }{2},\frac{\pi }{2}\right]$ and $\left[ \frac{\pi }{2},\frac{3\pi }{2}\right]$, otherwise, the magnetic field would reproduce an evolution loop effect, either closed or broken.

We shall remark that the trajectory smeared in the interval $\left[ -\frac{\pi }{2},\frac{\pi }{2}\right] \cup \left[ \frac{\pi }{2},\frac{3\pi }{2}\right]$ is not yet determined at this stage, which calls for a separate computer routine to integrate (Eq. 17) in the asymmetric interval where the initial time will be $t_0=-\frac{\pi }{2}$. That is, the integration of Eq. (17) will be split into two subintervals, each one with a definite pulse $\beta (t)$. The evolution process will start at the time $-\frac{\pi }{2}$ with the first pulse $\beta (t)$, then continuing the integration in the following subinterval at $\frac{\pi }{2}$ with the second pulse $\beta (t)$ and finishing at $\frac{3}{2\pi }$, in order to draw a congruent trajectory.

**Figure 4:**
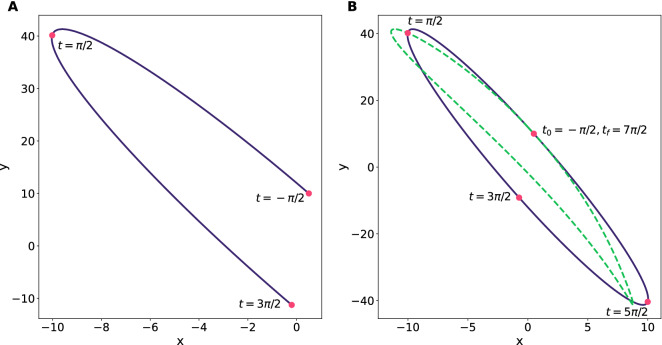
Semiclassical trajectories generated by the consecutive application of two polyharmonic pulses of the type (Eq. 29) over a particle with initial conditions $(x,y)=\left( \frac{1}{2}, 10\right)$ and $(p_x,p_y)=(-5,20)$. (**A**) The evolution process starts at $t_0=-\frac{\pi }{2}$ in terms of a polyharmonic amplitude (Eq. 29) defined by the parameters $b=2,c=-3$ finalising its application at $t=\frac{\pi }{2}$—where the amplitude vanishes. Immediately, at the very same time, the evolution process continues its development now in terms of the pulse defined by $b=\frac{9}{5},c=-\frac{7}{2}$ that will end its application at $t=\frac{3\pi }{2}$. The resulting operation attains a squeezing transformation with $\lambda _x=-0.403$ and $\lambda _y=-1.125$. (**B**) To invert the operation and recover the initial observables’s values, the second pulse has to be applied once again from $t=\frac{3\pi }{2}$ to $t=\frac{5\pi }{2}$, where the evolution process is now conducted by the first pulse during from $t=\frac{5\pi }{2}$ to $t=\frac{7\pi }{2}$. The dotted line represents the distorted evolution generated by the presence of an external harmonic force $\sin (t)$ in the direction *Ox*, even though note the original configuration is entirely recovered by reversing the operation.

Please note, as for the pulses in Fig. 3, the resulting control operation would be absent of any abrupt discontinuity even when it was regulated by the concatenation of two different fields, for example, the amplitudes $\beta (t)$ with solid and dashed curves can achieve a squeezing transformation in the fashion of (Eq. 20), whose actual trajectory in the *xOy* plane is plotted in Fig. 4(A) for a charged particle with initial conditions $(x,y)=\left( \frac{1}{2}, 10\right)$ and $(p_x,p_y)=(-5,20)$. The canonical observables of position are transformed as $X\rightarrow \lambda _xX$ and $Y\rightarrow \lambda _yY$ in agreement with (Eq. 20), our numerical computation indicates that such particle would be subject to a squeezing transformation with $\lambda _x=-0.403$ and $\lambda _y=-1.125$, meaning that the momenta have been amplified and compressed, respectively.

These effects, furthermore, can become significantly boosted by external driven forces either time-dependent or not. In such scenario the evolution process must account for the unperturbed Hamiltonian (Eq. 8) plus the external field. For instance, we want to consider a force aligned in the *Ox* direction, ${\mathbf {F}}=(F,0,0)$, leading to the perturbed Hamiltonian ${\widetilde{H}}(t)=H_\perp (t)+FX$, such that the corresponding evolution operator is constituted as ${\widetilde{U}}(t)=U(t)W(t)$, where *U*(*t*) is the unperturbed evolution operator, whereas *W*(*t*) represents the contribution of the perturbative force that obeys the differential equation

33 $$\begin{aligned} \frac{dW(t)}{dt} = iFX(t)W(t), \quad W(0)=\mathbbm{1}, \end{aligned}$$

where *X*(*t*) is the time-dependent observable fostered by the Heisenberg evolution (Eq. 10).

Even more, since the evolved observable *X*(*t*) is linear in the canonical variables, the commutators $[X(t'),X(t)]$ are reduced to numbers. The continuous Baker–Campbell–Hausdorff formula^42^ then implies that Eq. (33) has a solution of the form $e^{iF\int _0^tdt'\,X(t')}$ up to a phase factor $e^{i\phi }$, where $\phi$ is real. It follows that if $t=T$ is the loop period in the unperturbed evolution operator, then any initial coordinate ${\bar{X}}_j$ of the fuzzy centre (Eq. 4) will be reconstructed from the integral $\int _0^tdt'\,X(t')$, therefore ${\widetilde{U}}(T)=U(T)W(T)=e^{i\phi }e^{iTF{\bar{X}}_j}$. Suggesting that ${\bar{X}}_j$ will be unaffected by the transit of *W*(*t*), namely ${\widetilde{U}}^\dagger (T){\bar{X}}_j{\widetilde{U}}(T)=e^{-iTF{\bar{X}}_j}X_je^{iTF{\bar{X}}_j}={\bar{X}}_j$. However any other coordinate of the fuzzy centre could suffer a drift ${\widetilde{U}}^\dagger (T){\bar{X}}_{k}{\widetilde{U}}(T)={\bar{X}}_k-iTF[{\bar{X}}_j, \bar{X}_k]$, of course $j\ne k$.

We conclude that any fuzzy point $({\bar{X}}_j,\ldots ,{\bar{X}}_k)$ with noncommutative coordinates will result in a broken loop every time it is manoeuvred by an external force and the coordinates of its centre will drift in a transversal direction with respect to the force. On the contrary, if the fuzzy centre is such that its centre $[{\bar{X}}_j,{\bar{X}}_k]=0$, then at the end of the period *T* the point $({\bar{X}}_j,\ldots ,{\bar{X}}_k)$ will return to its initial value.

To illustrate our discussion, we have examined the inverted squeezing operation referred in Fig. 4(A) in the presence of a time-dependent perturbation $\sin (t)$ aligned in the *Ox* direction to distort the operational trajectory, see Fig. 4(B). As we mentioned before, the evolution process (17) first sweeps in the interval $t\in \left[ -\frac{\pi }{2},\frac{\pi }{2}\right]$ with a given polyharmonic pulse $\beta _1(t)$, and continues with a different $\beta _2(t)$ in the interval $t\in \left[ \frac{\pi }{2},\frac{3\pi }{2}\right]$. To invert the operation it is required, therefore, a second application of $\beta _2(t)$ with $t\in \left[ \frac{3\pi }{2},\frac{5\pi }{2}\right]$ and then a second application of $\beta _1(t)$ with $t\in \left[ \frac{5\pi }{2},\frac{7\pi }{2}\right]$. In our example, note that the perturbed evolution returns to its initial position after completing the period of application. Also the effect of the driven external force $\sin (t)$ in this case produces a set of transformations (Eq. 20) at $t=\frac{3\pi }{2}$ with $\lambda _x=3.92$ and $\lambda _y=-0.96$—unlike the unperturbed case, this time we have amplified the observable *X* and compressed *Y* at the cost of compressing $P_x$ and amplifying $P_y$.

**Figure 5:**
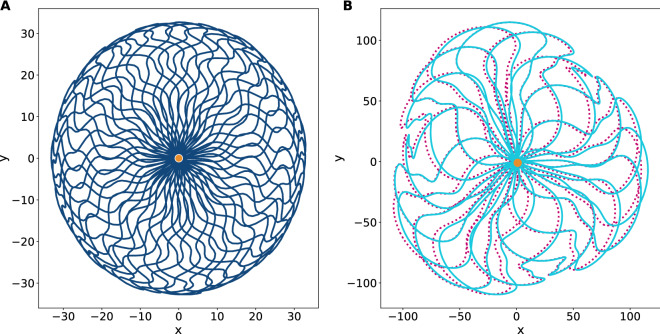
(**A**) Evolution loops performed via a polyharmonic field amplitude $\beta (t)$ characterised with the parameters $b=2,c=-5$, (see dotted pulse in Fig. 3). The charged particle has the initial conditions $(x,y)=(0,0)$ and $(p_x,p_y)=(10,5)$. The operation completes the loop after a period $T=52$. (**B**) Distorted evolution loop in terms of the polyharmonic field $\beta (t)$ with parameters $b=\frac{3}{2},c=-7$ and initial conditions $(x,y)=(1,-1)$, $(p_x,p_y)=(-30,30)$ subject to an external force of magnitude $F=\frac{1}{2}$ in the direction *Ox*. In solid (dotted) line, the unperturbed (perturbed) loop, closing after a period $T=39$. The perturbed loop exhibits a drifting effect in the opposite direction of the externally applied force. The dot at the centre of both figures represents the initial and final positions.

We have commented before that those polyharmonic pulses (Eq. 29) greater than zero convey the adequate oscillatory motion to produce squeezing operations, given that the corresponding matrix *h*(*t*) fulfils $|\Sigma |>2$. However, it has not been surveyed yet a more general type of pulses that can be either greater or less than zero, as the amplitude $\beta (t)$ with dotted line in Fig. 3, and whose associated matrix *h*(*t*) furnishes a stable motion in the region $|\Sigma |<2$. Accordingly, this class of pulses $\beta (t)$ does bring the alternative of evolution loop operations by merely implementing the programme outlined for biharmonic fields.

In this regard, please refer to the loops shown in Fig. 5. In panel A, we present a symmetric polyharmonic loop that is resistant to the application of an external potential. By *resistant* we mean that the loop is absolutely stable—as the examples shown in Fig. 2—and cannot be broken under the influence of forces coming from the outside, in fact, under these circumstances any solution of Eq. (33) will account as a phase factor. On the other hand, the loop in panel B has been deformed by an external field of constant magnitude (see the figure caption for details). It truly has a fuzzy point (Eq. 4) with noncommutative coordinates, then its fuzzy centre will suffer a drift in the transversal direction and eventually the loop could be broken.

## Discussion

Our discussion above is fundamentally focused on the time dependent family of matrices *h*(*t*) responsible for a number of quantum control operations, although our scheme does merely constitute an approximation. Usually this type of imperfections are not entirely disappointing: In Paul’s ion traps^6^, for instance, the propagation of the electromagnetic signals through the device’s interiors is usually disregarded due to the pretty small size of the trap. However, adopting a rigorous attitude, even those softly changing potentials sensed on the trap surfaces must produce field corrections starting from $\frac{1}{c}$ (post Newtonian) terms in the Einstein–Infeld–Hoffmann (EIH) approximation^43^. Below we are to briefly discuss a similar problem for softly changing magnetic fields $\beta (t)$ such as Eq. (12) or (29).

Throughout this report, we have been considering a time dependent, homogeneous magnetic field ${\mathbf {B}}(t,{\mathbf {x}})$ in a cylindric solenoid. It truly does not fulfil the Maxwell’s equations, yet it obeys a sequence of EIH approximations. To evaluate the conveyed errors, let us look for the exact time dependent vector potentials of a cylindrical solenoid in the form (Eq. 1):

34 $$\begin{aligned} {\mathbf {A}}(t,{\mathbf {x}}) = \frac{1}{2}B(t,r)\mathbf{n}\times \mathbf{x} = \frac{1}{2}B(t,r)\begin{pmatrix}-y\\ x \end{pmatrix}, \end{aligned}$$

where the magnetic field *B* instead of depending only on *t*, could also depend on the radius *r* of the solenoid’s cross section, and ${\mathbf {n}}$ is a unit vector in the field direction. To assure the relativistic sense of Eq. (34) we must assume $\square \mathbf{A}(t,{\mathbf {x}}) = \frac{4\pi }{c} \mathbf{J}(t,{\mathbf {x}}) = 0$, with the notation $\square = \frac{1}{c^2}\frac{\partial ^2}{\partial t^2}-\nabla ^2$ (the d’Alembert operator). As easily seen, the application of the Laplacian to the right hand side of Eq. (34) is equivalent to the acts of the operator $D = \frac{\partial ^2}{\partial r^2}+\frac{3}{r}\frac{\partial }{\partial r}$ to *B*(*t*, *r*) alone. Hence, $\square \mathbf{A}(t,{\mathbf {x}})$ means:

35 $$\begin{aligned} \left[ \frac{1}{c^2}\frac{\partial ^2}{\partial t^2}-D\right] B(t,r) = 0. \end{aligned}$$

The magnetic field *B*(*t*, *r*) must be analytic around *Oz*, then we preferably look for a solution in the form:

36 $$\begin{aligned} B(t,r) = B_0(t)+B_2(t)r^2+B_4(t)r^4+\cdots \end{aligned}$$

where $B_0(t) = B(t)$ is the homogeneous quasi-static approximation. Since $Dr^{2n}=4n(n+1)r^{2(n-1)}$, the substitution of Eq. (36) into Eq. (35) yields:

37 $$\begin{aligned} B_{2n}(t) = \frac{1}{4^n n! (n+1)!}\frac{1}{c^{2n}}\frac{\partial ^{2n}}{\partial t^{2n}}B(t). \end{aligned}$$

We now introduce a dimensionless time $\tau = \frac{t}{T}$, where *T* is some conventional time unit corresponding to the laboratory observation, and by writing Eq. (37) in terms of the time derivatives $\frac{\partial }{\partial \tau }$ one can reduce it to:

38 $$\begin{aligned} B(t,r) = B(t) + \frac{1}{8}\left( \frac{r}{cT}\right) ^2\frac{\partial ^2 B(t)}{\partial ^2 \tau } + \frac{1}{24}\left( \frac{r}{cT}\right) ^4\frac{\partial ^4 B(t)}{\partial ^4 \tau }+\cdots . \end{aligned}$$

We kept here the *B*(*t*) depending on the actual time *t*, in order to assure that all derivatives $\frac{\partial ^n}{\partial \tau ^n}B(t)$ will be expressed in magnetic field units. The curious property of this formula is the absence of terms proportional to $\frac{1}{c}$ —in fact, the field propagation law (Eq. 35) is solved exclusively in terms of extremely small contributions proportional to $\frac{1}{c^2}$. It suggests that the superpositions of delicate wave fronts running towards the solenoid centre create a good approximation of the quasi-static theory.

Even though, this does not explain how to create (or at least approximate) the first magnetic step *B*(*t*), to wake up the whole iterative series (Eq. 38). (That is, how to induce the homogeneous surface currents which do not depend on *z*, but depend on time in any desired way.)

In the static case, the magnetic field inside the solenoid is generated by a stationary current *I* circulating around the surface, i.e., $B = \frac{4\pi }{c}\frac{\Delta I}{\Delta z}$. The question is, how to produce the circulating currents depending on time, but homogeneous (independent from *z*) on every section of the surface? Should the solenoid was constructed as a single spiral wire around the cylindrical surface, connected at its both extremes to the potential difference $\Phi (t)$, then even a subtle change $\delta \Phi (t)$ would be propagated along the solenoid as a current pulse, creating a softly changing but *z*-dependent field, instead of the desired quasi-static *B*(*t*).

There is an alternative. One rather could consider a cylindrical surface of non-conducting material (e.g., glass) of radius *a*, charged uniformly with a surface density $\sigma$, so that each circular belt of 1cm of height contains a charge $a\sigma$. The experimental challenge is not extraordinary. Suppose the cylinder has a radius $a=20$cm, and it is rotating with an angular velocity $\omega = 1\text {s}^{-1}$ around its symmetrical axis aligned to *Oz* and has 1C of charge at every horizontal belt of 1cm, then inside the cylinder will be generated a homogeneous magnetic field $\mathbf{n} B$ of intensity:

39 $$\begin{aligned} B = \frac{4\pi }{c}\omega R\sigma \simeq 1.25\text {gauss}, \end{aligned}$$

at least in the post-post-Newtonian approximation. Hence, by employing the softly changing angular velocity $\omega \rightarrow \omega (t)$ one truly can generate the practically homogeneous magnetic field $\mathbf{n}B(t)$ of the quasi-static environment described by Eq. (38). Will such technique work?

As a remark, the actual operation time $\frac{t}{T} \rightarrow t$ given in Eq. (6) can be arbitrarily large (or short) and the fields (Eq. 7) arbitrarily strong (or weak). To form an idea of the real orders of magnitude required by our control operations, we shall present the Table 1 below.

To this aim, we have also decided to check the magnitude of the Abraham-Lorentz radiative force. While the ordinary force of the variable oscillator trajectory is simply ${\mathbf {F}}_\text {osc}(t)=m\ddot{{\mathbf {x}}}(t)$, the hypothetical radiative force is expressed as ${\mathbf {F}}_\text {rad}(t) = m\gamma \dddot{{\mathbf {x}}}(t)$, where $\gamma =\frac{2}{3}\frac{e^2}{mc^3}$ is the particle dependent characteristic time. Using now the definitions of dimensional quantities (Eq. 6) we can compare the magnitudes of the conventional and radiative forces for the squeezing operations (see the last row in Table 1) finding the radiative ones extremely small albeit slowly increasing as *T* decreases (higher frequencies).

**Table 1:** Physical conditions (in cgs) to achieve the squeezing transformations $\lambda _x=-0.43$ and $\lambda _y=-1.125$ on a proton moving inside a cylindrical solenoid. The reported quantities have been calculated regarding the example in Fig. 4. The physical magnitudes of *q*, *p* and *v*, with $q=x=y$ and $p=p_x=p_y$ correspond to the dimensionless values $x'=y'=p'_x=p'_y=1$ according to Eq. (6). Note how the incredibly modest strength of the magnetic field grows as the operation time *T* becomes shorter, to form an idea, a control operation of $T=10^{-2}$s. would require half the strength of Earth’s magnetic field on the equator. In the last row it is reported the average ratio of the Abraham-Lorentz radiative force to the time-dependent oscillator forces, at different operation intervals *T*.

*T* (s)	$10^{-2}$	1	$10^2$
*q* (cm)	2.5$\times 10^{-3}$	2.5$\times 10^{-2}$	2.5$\times 10^{-1}$
*p* (g cm $\text {s}^{-1}$)	4.2$\times 10^{-25}$	4.2$\times 10^{-26}$	$4.2\times 10^{-27}$
*v* (cm $\text {s}^{-1}$)	2.5$\times 10^{-1}$	2.5$\times 10^{-2}$	2.5$\times 10^{-3}$
$B_{\text {max}}$ (gauss)	1.5$\times 10^{-2}$	1.5$\times 10^{-4}$	1.5$\times 10^{-6}$
$F_\text {rad}/F_\text {osc}$	5$\times 10^{-25}$	5$\times 10^{-27}$	5$\times 10^{-29}$

Our preliminary evaluations become valid whenever the physical size of the solenoid is realisable. In many laboratories the techniques to cool and trap ions are adequate to hardly study the atomic structure but insufficient for wider purposes, since the time dependent oscillator potential is created only in a strictly local scale, such is the case of a quadrupole trap whose immediate vicinity from the central axis is conformed, in some cases, just by four metal bars^15^.

In our calculations we have assumed pure states, evolving under the influence of slowly changing external fields, without taking corrections for traces of matter across the ion traps or solenoids. Moreover, we have neglected the possible packet reflection or absorption by the laboratory walls. Suggesting an apparatus whose dimensions make any of these considerations to become insignificant. Actually, in case that particle would be absorbed by the (surface) walls, the problem leads to the fundamental question of the time operator which persists without a truly convincing solution even in case of flat surfaces. We thus hope that for wide traps, our proposal brings something of interest to the quantum control theory.

As well, we have noticed that in works^41,44^ using the Ermakov-Milne invariants the operations are supposed to be faster that the times given here. In that regard, our contribution is slightly different: Its main goal is the use of a simple Toeplitz algebra to obtain the exact solutions (Eq. 29) whereas other approaches use the suggestive idea of *frictionless driving*, which transports the states without changing the eigenvalues of certain invariants. But could the more general variational methods be applied at the level of a $\theta (t)$ function? The question remains open.
