# Publisher Correction: Quantum control operations with fuzzy evolution trajectories based on polyharmonic magnetic fields

**DOI:** 10.1038/s41598-021-96665-1

**Published:** 2021-08-30

**Authors:** Jesús Fuentes

**Affiliations:** División de Ciencias e Ingenierías, Departamento de Física, Universidad de Guanajuato, Loma del Bosque 103, 37150 León, Guanajuato Mexico

Correction to: *Scientific Reports* 10.1038/s41598-020-79309-8, published online 17 December 2020

The original version of this Article contained a repeated error, where the Blackboard Bold 1 symbol did not display correctly in Equations 9, 17, 25, 26, and 33, and in the Results section.

In addition, in the Introduction,

“Note that if we define $\rho^2=(X - \bar{X})^2 + (Y - \bar{X})^2$, then $\pi \rho ^2=\frac{2\pi }{m\omega _c^2}H$ can be interpreted as a fuzzy surface spanned by the transversal orbits.”

now reads:

“Note that if we define $\rho^2=(X - \bar{X})^2 + (Y - \bar{Y})^2$, then $\pi \rho ^2=\frac{2\pi }{m\omega _c^2}H$ can be interpreted as a fuzzy surface spanned by the transversal orbits.”

Finally, in the Results section, under the subheading ‘Polyharmonic fields: exact operations’,

“However any other coordinate of the fuzzy centre could suffer a drift $\widetilde{U}^\dagger(T)\bar{X}_{k}\widetilde{U}(T) = \bar{X}_k - iTF[\bar{X}_j, \hat{X}_k]$, of course $j\ne k$.”

now reads:

“However any other coordinate of the fuzzy centre could suffer a drift $\widetilde{U}^\dagger(T)\bar{X}_{k}\widetilde{U}(T) = \bar{X}_k - iTF[\bar{X}_j, \bar{X}_k]$, of course $j \neq k$.”

The original Article has been corrected.

